# Vitamin D deficiency is associated with a worse prognosis in metastatic melanoma

**DOI:** 10.18632/oncotarget.14316

**Published:** 2016-12-28

**Authors:** Dmitriy Timerman, Melissa McEnery-Stonelake, Cara J Joyce, Vinod E Nambudiri, F Hodi Stephen, Elizabeth B Claus, Nageatte Ibrahim, Jennifer Y Lin

**Affiliations:** ^1^ Harvard-MIT Health Sciences and Technology (HST), Harvard Medical School, Boston, MA, USA; ^2^ Department of Dermatology, University of Alabama at Birmingham, Birmingham, AL, USA; ^3^ Department of Biostatistics, Tulane University School of Public Health and Tropical Medicine, New Orleans, LA, USA; ^4^ Department of Dermatology, Brigham and Women's Hospital, Harvard Medical School, Boston, MA, USA; ^5^ Melanoma Program, Dana Farber Cancer Institute, Harvard Medical School, Boston, MA, USA; ^6^ Department of Neurosurgery, Brigham and Women's Hospital, Harvard Medical School, Boston, MA, USA; ^7^ Merck Research Laboratories, Clinical Oncology, North Wales, PA, USA

**Keywords:** vitamin D deficiency, melanoma, 25(OH)D3

## Abstract

Vitamin D deficiency (≤20 ng/mL) is associated with an increased incidence and worse prognosis of various types of cancer including melanoma. A retrospective, single-center study of individuals diagnosed with melanoma from January 2007 through June 2013 who had a vitamin D (25(OH)D3) level measured within one year of diagnosis was performed to determine whether vitamin D deficiency and repletion are associated with melanoma outcome. A total of 409 individuals diagnosed with histopathology-confirmed melanoma who had an ever measured serum 25(OH)D3 level were identified. 252 individuals with a 25(OH)D3 level recorded within one year after diagnosis were included in the study and the individual and melanoma characteristics such as age, sex, Breslow thickness, ulceration, stage, mitotic rate, and LDH were obtained from the medical record. A worse melanoma prognosis was associated with vitamin D deficiency (P=0.012), higher stage (P<0.001), ulceration (P=0.001), and higher mitotic rate (P=0.001) (HR 1.93, 95% CI 1.15-3.22). In patients with stage IV metastatic melanoma, vitamin D deficiency was associated with significantly worse melanoma-specific mortality (adjusted HR 2.06, 95% CI 1.10-3.87). Patients with metastatic melanoma who were initially vitamin D deficient and subsequently had a decrease or ≤20 ng/mL increase in their 25(OH)D3 concentration had significantly worse outcomes (HR 4.68, 95% CI 1.05-20.88) compared to non-deficient patients who had a >20 ng/mL increase. Our results suggest that initial vitamin D deficiency and insufficient repletion is associated with a worse prognosis in patients with metastatic melanoma.

## INTRODUCTION

Melanoma is a commonly fatal skin cancer with an estimated 9,710 deaths out of a projected 76,100 cases diagnosed in the United States in 2014. [1] The rising incidence of melanoma has driven increased interest in the field. Of all skin cancers, it accounts for 75% of skin cancer deaths and has increased in incidence at a rate of 3.1% per year from 1992 to 2004. [2] In the United States, lifetime risk of developing melanoma based on 2010 risk estimates is 1 in 39 for Caucasian men and 1 in 58 for Caucasian women. [3]

One area of increased investigation and debate is the relationship between vitamin D and melanoma. [4–6] Recent studies have suggested an association between serum 25-hydroxyvitamin D3 levels (25(OH)D3), incidence, and prognosis of various cancers including colorectal and pancreatic cancer. [7, 8] Analysis of a German cohort of patients with melanoma suggested that lower serum 25(OH)D3 concentrations are associated with an increased risk for melanoma, greater Breslow thickness, and worse overall survival. [9] A recent prospective study on the prognostic value of vitamin D levels, demonstrated that 25(OH)D3 level variation during follow-up, but not the level at diagnosis, was an independent melanoma prognostic marker. [10] However, the relationship between vitamin D and melanoma risk is controversial with other studies reporting either no association between serum vitamin D levels and melanoma mortality, [11] or an increased melanoma incidence in individuals with high vitamin D levels. [12, 13] Complicating the association between vitamin D and melanoma is the biosynthetic mechanism of active vitamin D. In particular, UVB exposure confounds the relationship between serum vitamin D concentrations and melanoma risk by increasing both simultaneously. The finding that sun exposure may be associated with increased survival in patients with melanoma [14] could therefore be partly explained if vitamin D has a protective role in patients with melanoma. [15]

Studies of vitamin D deficiency repletion have been of particular interest given the ease with which supplementation can be implemented. In a post-hoc analysis of the Women's Health Initiative randomized controlled trial, a 57% reduction (P=0.04) in melanoma incidence was observed in postmenopausal women with a self-reported history of non-melanoma skin cancer who took 1000 mg of calcium and 400 IU of oral vitamin D supplementation daily compared to those receiving placebo. [16] Vitamin D supplementation, however, was not associated with reduced melanoma risk in the entire study group of 36,282 women.

We performed a retrospective study in 252 patients with a melanoma diagnosis referred to a tertiary cancer center with at least one serum vitamin D measurement within one year after diagnosis to test the hypothesis that the initial serum 25(OH)D3 concentrations and the change in 25(OH)D3 levels were associated with melanoma prognosis.

## RESULTS

**Figure 1:**
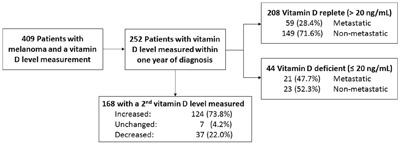
Patient Selection for Study Eligibility

A total of 252 patients with melanoma and at least one serum 25-hydroxyvitamin D3 measurement within one year after diagnosis were identified (Figure 1). Patient characteristics grouped by survival are summarized in Table 1. Patients who died were significantly older (P=0.007), more likely to have an LDH greater than 240 U/L (P<0.001), ulceration on pathology (P=0.001), higher mitotic rate (P=0.001), vitamin D deficiency (≤20 ng/mL, P=0.012), and higher stage (P<0.001) compared to those who were alive or lost to follow-up at the end of the study.

**Table 1:** Participant Characteristics by Survival Status

	OverallN=252	DiedN=80	AliveN=172	p-value*
Age, mean (SD)	55.4 (14.7)	59.0 (13.4)	53.7 (14.9)	0.007
Age, n (%)				
≤ 50 yrs	93 (36.9)	22 (27.5)	71 (41.3)	0.035
> 50 yrs	159 (63.1)	58 (72.5)	101 (58.7)	
Sex, n (%)				
Female	108 (42.9)	28 (35.0)	80 (46.5)	0.086
Male	144 (57.1)	52 (65.0)	92 (53.5)	
Breslow's depth^, n (%)				
< 1 mm	43 (25.0)	3 (10.7)	40 (27.8)	0.112
1-4 mm	91 (52.9)	16 (57.1)	75 (52.1)	
> 4 mm	38 (22.1)	9 (32.1)	29 (20.1)	
LDH, n (%)				
≤ 240 U/L	200 (90.5)	60 (76.9)	140 (97.9)	<0.001
> 240 U/L	21 (9.5)	18 (23.1)	3 (2.1)	
Ulceration, n (%)				
Absent	126 (65.6)	24 (47.1)	102 (72.3)	0.001
Present	66 (34.4)	27 (52.9)	39 (27.7)	
Mitotic rate, n (%)				
0	19 (9.7)	2 (3.9)	17 (11.7)	0.001
1-4	81 (41.3)	13 (25.5)	68 (46.9)	
> 4	96 (49.0)	36 (70.6)	60 (41.4)	
Vitamin D, n (%)				
> 20 ng/mL	208 (82.5)	59 (73.8)	149 (86.6)	0.012
≤ 20 ng/mL	44 (17.5)	21 (26.3)	23 (13.4)	
Stage, n (%)				
0	6 (2.4)	0 (0.0)	6 (3.5)	<0.001
1	38 (15.1)	3 (3.8)	35 (20.3)	
2	48 (19.0)	4 (5.0)	44 (25.6)	
3	80 (31.7)	21 (26.3)	59 (34.3)	
4	80 (31.7)	52 (65.0)	28 (16.3)	

N=60 missing ulceration; N=56 missing mitotic rate; N=31 missing LDH.

^N=80 metastatic not included.

*p-value from t-tests for continuous variables, chi-square test for nominal variables.

Patient characteristics grouped by vitamin D levels are shown in Table 2. Patients with vitamin D deficiency (≤ 20 ng/mL) were significantly more likely to have higher stage melanoma (P=0.010) and die during follow-up (P=0.012) compared to patients with vitamin D levels > 20 ng/mL.

**Table 2:** Participant Characteristics by Vitamin D Level

	OverallN=252	≤ 20 ng/mLN=44	> 20 ng/mLN=208	p-value*
Age, mean (SD)	55.4 (14.7)	53.4 (15.6)	55.8 (14.5)	0.312
Age, n (%)				
≤ 50 yrs	93 (36.9)	22 (50.0)	71 (34.1)	0.048
> 50 yrs	159 (63.1)	22 (50.0)	137 (65.9)	
Sex, n (%)				
Female	108 (42.9)	20 (45.5)	88 (42.3)	0.702
Male	144 (57.1)	24 (54.6)	120 (57.7)	
Breslow's depth^, n (%)				
< 1 mm	43 (25.0)	6 (26.1)	37 (24.8)	0.846
1-4 mm	91 (52.9)	11 (47.8)	80 (53.7)	
> 4 mm	38 (22.1)	6 (26.1)	32 (21.5)	
LDH, n (%)				
≤ 240 U/L	200 (90.5)	33 (84.6)	167 (91.8)	0.223
> 240 U/L	21 (9.5)	6 (15.4)	15 (8.2)	
Ulceration, n (%)				
Absent	126 (65.6)	21 (75.0)	105 (64.0)	0.258
Present	66 (34.4)	7 (25.0)	59 (36.0)	
Mitotic rate, n (%)				
0	19 (9.7)	2 (6.9)	17 (10.2)	0.673
1-4	81 (41.3)	14 (48.3)	67 (40.1)	
> 4	96 (49.0)	13 (44.8)	83 (49.7)	
Stage, n (%)				
0	6 (2.4)	1 (2.3)	5 (2.4)	0.010
1	38 (15.1)	2 (4.5)	36 (17.3)	
2	48 (19.0)	3 (6.8)	45 (21.6)	
3	80 (31.7)	17 (38.6)	63 (30.3)	
4	80 (31.7)	21 (47.7)	59 (28.4)	
Died				
Yes	80 (31.7)	21 (47.7)	59 (28.4)	0.012
No	172 (68.3)	23 (52.3)	149 (71.6)	

N=60 missing ulceration; N=56 missing mitotic rate; N=31 missing LDH.

^N=80 metastatic not included.

*p-value from t-tests for continuous variables, chi-square test for nominal variables.

In multivariate analysis, worse outcomes were observed in patients with vitamin D deficiency (HR = 1.939 % 11.15-3.22) as shown in Table 3. In patients with non-metastatic melanoma, we did not observe significantly worse outcomes with increased age, male sex, vitamin D deficiency, elevated LDH level, or presence of ulceration (data not shown). In patients with stage IV metastatic melanoma, vitamin D deficiency was associated with a hazard ratio of 2.06 (95% CI 1.10-3.87) after adjusting for age, gender, and LDH level (Table 4). Supplementary Figure 1 shows the survival curve for metastatic melanoma in patients with and without vitamin D deficiency.

**Table 3:** Unadjusted and Adjusted Hazard Ratios for Death from Melanoma associated with Vitamin D level

	Unadjusted HR(95% CI)	Adjusted HR(95% CI)
Age (yrs)		
≤ 50	1 (Reference)	1 (Reference)
> 50	1.63 (0.99, 2.67)	1.56 (0.94, 2.59)
Sex		
Female	1 (Reference)	1 (Reference)
Male	1.69 (1.07, 2.68) †	1.49 (0.93, 2.37)
Vitamin D (ng/mL)		
> 20	1 (Reference)	1 (Reference)
≤ 20	2.08 (1.27, 3.43) ‡	1.93 (1.15, 3.22) †
Stage		
0-2	1 (Reference)	1 (Reference)
3	3.84 (1.63, 9.04) ‡	3.45 (1.45, 8.19) ‡
4	16.71 (7.55, 36.98) ‡	15.07 (6.77, 33.55) ‡

† p<0.05;

‡ p<0.01.

Model N=252.

**Table 4:** Unadjusted and Adjusted Hazard Ratios for Death from Melanoma associated with Vitamin D level in Patients with Metastatic Melanoma

	Unadjusted HR(95% CI)	Adjusted HR(95% CI)
Age (yrs)		
≤ 50	1 (Reference)	1 (Reference)
> 50	1.38 (0.75, 2.56)	1.28 (0.69, 2.39)
Sex		
Female	1 (Reference)	1 (Reference)
Male	2.00 (1.09, 3.67) †	1.75 (0.92, 3.31)
Vitamin D (ng/mL)		
> 20	1 (Reference)	1 (Reference)
≤ 20	2.25 (1.23, 4.11) ‡	2.06 (1.10, 3.87) †
LDH (U/L)		
≤ 240	1 (Reference)	1 (Reference)
> 240	2.18 (1.18, 4.02) †	1.66 (0.87, 3.18)

† p<0.05;

‡ p<0.01.

N=2 missing LDH.

Model N=78.

**Figure 2:**
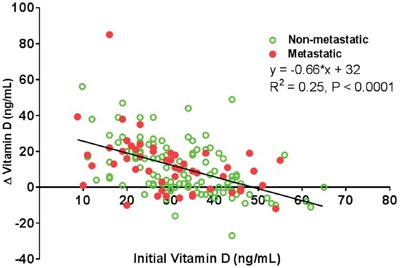
Comparison between Initial and Change in Vitamin D Levels Lower initial 25(OH)D3 levels were associated with a higher increase in vitamin D on subsequent measurements (P<0.0001). A linear regression (change in vitamin D level = -0.66*(initial vitamin D level) + 32, R^2^ = 0.25) was fit to the entire dataset (N=168) of individuals who had an initial 25(OH)D3 level measured within one year after diagnosis and also had a 25(OH)D3 measurement following supplementation. Individuals with non-metastatic disease are color-coded with empty green circles, while those with metastatic disease are color-coded with filled red circles. Statistically significant linear associations were obtained for the non-metastatic (R^2^ = 0.21, P=0.0006) and metastatic groups (R^2^ = 0.26, P<0.0001) separately as well.

Lower initial 25(OH)D3 levels were linearly associated with a significantly greater increase in vitamin D on a subsequent measurement (P<0.0001) in both patients with metastatic and non-metastatic disease (Figure 2). Patients with metastatic melanoma who were vitamin D deficient at presentation or on the subsequent measurement had significantly worse outcomes (HR 2.26, 95% CI 1.23-4.17) compared to those who were vitamin D replete at both time points. In a multivariate analysis of patients with non-metastatic melanoma, the hazard ratios for changes in 25(OH)D3 levels were not statistically significant (data not shown). In a multivariate analysis adjusting for age, sex, and LDH, patients with metastatic melanoma who were initially vitamin D deficient (≤20 ng/mL) and who subsequently had a decrease or ≤20 ng/mL increase in their 25(OH)D3 levels had significantly worse outcomes (HR 4.68, 95% CI 1.05-20.88) compared to vitamin D replete patients who had a >20 ng/mL increase on a subsequent measurement (Supplementary Table 1). LDH above 240 U/L was associated with a significantly increased HR of 2.48 (95% CI 1.10-5.58). There was no significant association between seasons and vitamin D concentrations in our cohort (Supplementary Table 2).

## DISCUSSION

Vitamin D deficiency has traditionally been associated with pathologies of the bone including rickets, osteomalacia, osteoporosis, and fractures. Recently, there has been increased interest in the relationship between vitamin D and cancer. In a meta-analysis of 19 randomized clinical trials, an estimated 6% reduction in colorectal cancer risk was observed for each 4 ng/mL increase in pre-diagnosis serum vitamin D concentration. [17] In another meta-analysis of 18 randomized clinical trials, vitamin D supplementation was found to significantly reduce all-cause mortality. [18] For patients with melanoma, several studies suggest that vitamin D may delay melanoma recurrence and improve overall prognosis. [19, 20] The use of vitamin D as an adjuvant therapy for patients with melanoma is being actively explored. [21]

In our study, vitamin D deficiency was seen more commonly in patients with higher stage melanoma and correlated with a worse outcome, which is in agreement with other studies. [9, 20] Although, we did not see a significant correlation between vitamin D levels and Breslow's depth as suggested in other studies, [19, 20] we did observe a non-significant trend of worse outcomes with increased depth (<1mm: HR 1, Reference; 1-4mm: HR 1.82, 95% CI 0.41-8.15; >4 mm: HR 2.18, 95% CI 0.41-11.56) after adjusting for age, sex, vitamin D, LDH, and ulceration. A larger sample size may be necessary to observe this relationship. We also did not see a significant correlation between vitamin D levels and survival and non-metastatic melanoma which may in part be due to the low number of early stage melanomas in our analyses.

Currently, there is no standard recommendation regarding measurement or repletion of vitamin D for patients with melanoma. In our practice, 25(OH)D3 levels are commonly measured in this patient population and vitamin D supplementation is suggested, especially for patients who are deficient or who may initiate aggressive sun avoidance and protection following diagnosis. For this study, we did not have a mechanism to track or confirm if and to what extent a patient was supplementing vitamin D. However, given that we counseled all patients to stay out of the sun and take vitamin supplementation, we assume that the observed increase in vitamin D levels in 124 (73.8%) of the 168 patients with two vitamin D measurements was primarily due to supplementation.

Patients with metastatic melanoma who were initially vitamin D deficient and did not have an increase in 25(OH)D3 levels of >20 ng/mL had a worse prognosis compared to those who were vitamin D replete and had an increase of >20 ng/mL. Neither the patients who were initially vitamin D replete and had a decrease or ≤20 ng/mL increase nor the ones who were initially vitamin D deficient and subsequently had a >20 ng/mL increase had significantly worse outcomes compared to the patients who were vitamin D replete and had an increase of >20 ng/mL. This suggests that metastatic melanoma patients with vitamin D deficiency who are unable to raise their 25(OH)D3 levels have a worse prognosis compared to those who are vitamin D replete initially or are vitamin D deficient, but are able to markedly (>20 ng/mL) increase their 25(OH)D3 levels. A recent study found that variation in 25(OH)D3 levels, including both large increases and decreases, was an independent prognostic marker in patients with melanoma. [10] Stratification by the initial 25(OH)D3 level is important when comparing changes in vitamin D since as shown in Figure 2: the patients who have large increases in 25(OH)D3 levels tend to be the ones with a low starting level. Conversely, the patients who have a decrease in 25(OH)D3 levels tend to be the ones with a high starting level. Our results suggest that both initial vitamin D deficiency and inadequate repletion, when normalized by initial 25(OH)D3 levels, portend a worse prognosis in patients with metastatic melanoma. Saiag et al emphasize that the initial 25(OH)D3 level was not an independent melanoma prognostic marker when adjusted for other prognostic variables. Our results support this finding in the non-metastatic patient population. Currently, LDH is the only serum marker to offer prognostic information in metastatic melanoma patients; however in our cohort, low 25(OH)D3 levels had more prognostic significance than LDH. Statistically significant relationships between changes in 25(OH)D3 levels and outcome were not observed in the non-metastatic melanoma group.

There are several potential explanations for the results observed in this study. Serum 25(OH)D3 has been associated with robust immune response and better prognosis in colon cancer. [22] Serum 25(OH)D3 may likewise serve as a marker of adequate immune response in patients with metastatic melanoma. Alternatively, low serum vitamin D levels may represent less chronic sun exposure and a propensity for sunburns, and may be a surrogate marker of an individual who is at higher risk of developing melanoma. Serum vitamin D levels may also represent a marker for another unknown variable or effect that is causally related to melanoma prognosis. For instance, it has been established that vitamin D levels are lower in obese and physically inactive adults. [23] Metastatic spread may also induce vitamin D deficiency secondary to disease burden or subsequent sun avoidance. The effect of emerging treatments, such as immunotherapy, for metastatic melanoma on vitamin D levels is not well-known; however, such treatments may also influence vitamin D levels.

Our finding of a significantly worse survival in men is in agreement with other studies which also show that men with melanoma have a worse prognosis compared to women, even when adjusted for multiple prognostic factors including age, Breslow thickness, and ulceration. [24] Compared to women, men over 65 with melanoma have increased mortality rate, but the biologic factors on why this may be true remain unclear. [25] In our cohort, patients older than 50 years were less likely to be vitamin D deficient compared to those who were younger than 50 years; however, this result was borderline statistically significant (P=0.048). This may be due to increased awareness of bone health in older age and subsequently increased likelihood of taking supplementation prior to melanoma diagnosis.

The underlying mechanism for vitamin D deficiency in patients with metastatic melanoma is important for understanding if a vitamin D-based therapy could improve outcomes. Although whether or not vitamin D supplementation is a viable method to improve melanoma outcomes is not established in this study, measurement of vitamin D levels and correction of vitamin D deficiency should be considered in patients with metastatic melanoma to at a minimum reduce the known adverse musculoskeletal effects of vitamin D deficiency. Large-scale prospective studies in patients with a melanoma diagnosis who receive vitamin D supplementation are necessary to determine if a causal relationship exists and if supplementation could be beneficial.

The present study had several limitations. Although more than 400 patients with a melanoma diagnosis and a 25(OH)D3 measurement were initially identified, only 252 had a 25(OH)D3 level measured within one year after diagnosis. In our institution, 25(OH)D3 levels were checked after acute procedures such as excision were completed and may not have been representative of the 25(OH)D3 level at initial presentation or diagnosis. The low number of cases with pre-diagnosis 25(OH)D3 measurements precluded analysis of pre-diagnosis levels. Similarly, the number of patients with vitamin D deficiency on a second measurement was too low to perform subgroup analysis. The rate of vitamin D supplementation prior to or following melanoma diagnosis was not incorporated into our analysis as it was not feasible to verify the sources, amount, or frequency with which patients were adhering to vitamin D supplementation recommendations. Whether the measured 25(OH)D3 levels and subsequent changes were secondary to supplementation or other sources cannot be definitively determined. Our results identify associations between 25(OH)D3 levels and melanoma prognosis, although cannot specify what therapy could be used to affect 25(OH)D3 levels. The staging of disease in this report was based on the results on the initial clinical workup. Patients with non-metastatic disease who progressed to stage IV disease after one year where classified according to their original stage, although the effect of rapid disease progression may have an effect on 25(OH)D3 levels and capacity for repletion. Lastly, the population studied was limited to one tertiary cancer center in the Northeastern United States and therefore the effect of sun exposure on melanoma risk and vitamin D levels may not be generalizable to regions with different annual amounts of sunlight. In addition, the population of melanoma patients was skewed to later stage melanomas and therefore the earlier stage melanomas were not adequately represented.

Our results support the hypothesis that vitamin D deficiency and insufficient repletion is associated with a worse prognosis in individuals recently diagnosed with melanoma. The retrospective basis of this study limits a causal explanation for these results, but strongly suggests the need for future clinical trials evaluating whether vitamin D supplementation is beneficial for patients with metastatic melanoma.

## MATERIALS AND METHODS

### Patient population

This retrospective study was performed in agreement and accordance with IRB approval. An institution-specific oncology database of patients referred to our tertiary cancer center located in the Northeastern United States was used to identify patients with a diagnosis of melanoma and extract patient data including date of birth, date of death from any cause, date of melanoma diagnosis, and sex. All patient data were confirmed using the electronic medical record system and additional information including date of last contact with the patient were obtained.

Individuals diagnosed with histopathology-confirmed melanoma including both melanoma-in-situ and metastatic melanoma from January 2007 through June 2013 who had an ever measured vitamin D level in our system were included (n=409). Patient records were anonymized and de-identified prior to analysis. Serial serum vitamin D (25(OH)D3) concentrations and the associated date of draw were obtained from the electronic medical record on all patients. Of note, several clinicians in our practice are interested in trending and repleting vitamin D levels, and vitamin D levels were frequently drawn after initial treatments. Patients with vitamin D deficiency were advised to take 50,000 IU vitamin D3 per week for 8 weeks and then continue on 4,000 IU per week.

252 individuals with a 25(OH)D3 level recorded within one year after diagnosis were included in the study. For comparison of the effect of changes in 25(OH)D3 levels on survival, analysis included those individuals with a 25(OH)D3 level recorded within one year after diagnosis and who had a recorded subsequent 25(OH)D3 level at any time. This subgroup included 168 individuals. Survival analysis based on subgroups of interest including metastatic spread, Breslow's depth, age, and sex were also performed. Patients were considered to have metastatic stage IV disease if distant metastasis were present following a standard staging workup of the initial diagnosis. Breslow's depth, ulceration, and mitotic rate were not available for all patients, especially when a primary lesion was not identified. The LDH value recorded within one year of diagnosis were used.

The medical histories of all patients with melanoma and an ever measured 25(OH)D3 level were obtained and reviewed to identify demographic information and clinical characteristics including gender, skin color, date of birth, date of melanoma-specific death, age at diagnosis, and Breslow thickness. The last patient note as of March 2015 was used for the final patient contact.

### Statistical analysis

The study population was divided based on groups of interest including those with vitamin D deficiency (≤20 ng/mL) and those without (>20 ng/mL), as well as gender, and stage. The 20 ng/mL for vitamin D deficiency was based on current practice guidelines from the Endocrine Society. P-values from t-tests were used for continuous variables and from chi-square tests for nominal variables. Hazard ratios and 95% confidence intervals were calculated using Mantel-Haenszel method. Sample size did not allow for tests of interactions of vitamin D with important covariates such as stage. A P-value of <0.05 was considered statistically significant. The Kruskal-Wallis test was used to determine if the median 25(OH)D3 concentration varied by season. Statistical analyses and figure generation was performed using SAS 9.4 (Cary, NC) and GraphPad Prism (Version 6.05, GraphPad Software, Inc., La Jolla, CA, USA).

## SUPPLEMENTARY MATERIALS FIGURE AND TABLES


