# Supplementary material for: Vitamin D deficiency is associated with a worse prognosis in metastatic melanoma
Source: Oncotarget. 2016 Dec 28;8(4):6873–82. doi: 10.18632/oncotarget.14316 (PMC5351676; doi:10.18632/oncotarget.14316)
Supplement: Supplementary file 1 [file oncotarget-08-6873-s001.pdf]

Vitamin D deficiency is associated with a worse prognosis in metastatic melanoma

SUPPLEMENTARY FIGURE AND TABLES

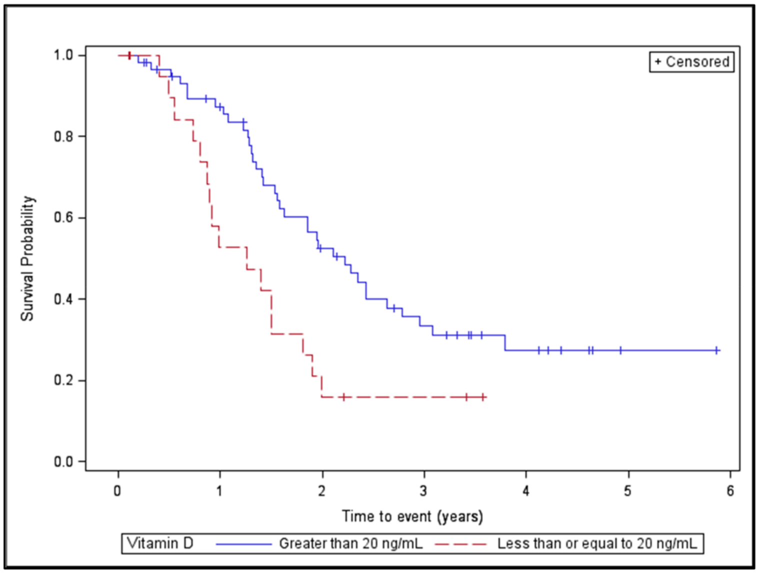

Supplementary Figure 1: Survival Curve for Patients with Metastatic (Stage IV) Melanoma grouped by Presence of Initial Vitamin D Deficiency.

Supplementary Table 1: Unadjusted and Adjusted Hazard Ratios for Death from Melanoma associated with Vitamin D level in Patients with Metastatic Melanoma

	Metastatic	
	**Unadjusted HR (95% CI)**	**Adjusted HR (95% CI)**
Age (yrs)		
≤ 50	1 (Reference)	1 (Reference)
> 50	1.00 (0.50, 2.02)	1.29 (0.60, 2.78)
Sex		
Female	1 (Reference)	1 (Reference)
Male	1.69 (0.82, 3.47)	1.56 (0.74, 3.29)
LDH (U/L)		
≤ 240	1 (Reference)	1 (Reference)
> 240	2.43 (1.15, 5.12) †	2.48 (1.10, 5.58) †
Vitamin D (ng/mL)		
Initial Vitamin D > 20, > 20 increase	1 (Reference)	1 (Reference)
Initial Vitamin D > 20, decrease or ≤ 20 increase	1.21 (0.36, 4.08)	0.91 (0.26, 3.21)
Initial Vitamin D ≤ 20, > 20 increase	1.70 (0.41, 7.13)	1.09 (0.24, 4.96)
Initial Vitamin D ≤ 20, decrease or ≤ 20 increase	5.18 (1.26, 21.38) †	4.68 (1.05, 20.88) †

†$p<0.05$.
N=2 missing LDH.
Model N=51.
Alternative adjusted hazard ratios: Vitamin D ≤ 20 ng/mL at either time point: 2.26 (1.23, 4.17) †.
Sample size:

Initial Vitamin D > 20, > 20 increase	(N=5)
Initial Vitamin D > 20, decrease or ≤ 20 increase	(N=32)
Initial Vitamin D ≤ 20, > 20 increase	(N=7)
Initial Vitamin D ≤ 20, decrease or ≤ 20 increase	(N=7)

Supplementary Table 2: Vitamin D by Season

	Winter (N=80, 31.7%)	Spring (N=61, 24.2%)	Summer (N=52, 20.6%)	Fall (N=59, 23.4%)
Median (IQR) Vitamin D	29.0 (15.5)	27.0 (18.0)	31.5 (12.0)	30.0 (16.0)
Vitamin D (ng/mL)	N (%)			
≤ 20	13 (16.3)	14 (23.0)	5 (9.6)	12 (20.3)
(20-40]	51 (63.8)	34 (55.7)	36 (69.2)	34 (57.6)
> 40	16 (20.0)	13 (21.3)	11 (21.2)	13 (22.0)

There are no significant differences in median vitamin D concentration by season (p=0.650 by Kruskal-Wallis test). Season is not associated with vitamin D concentration (p=0.630 by chi-square test).
